# Edible marijuana and cycle ergometer exercise

**DOI:** 10.3389/fphys.2022.1085822

**Published:** 2022-12-05

**Authors:** Taylor Russell Ewell, Matthew Charles Bomar, Kieran Shay Struebin Abbotts, Hannah Michelle Butterklee, Gregory P. Dooley, Christopher Bell

**Affiliations:** ^1^ Department of Health and Exercise Science, Colorado State University, Fort Collins, CO, United States; ^2^ Department of Environmental and Radiological Health Sciences, Colorado State University, Fort Collins, CO, United States

**Keywords:** cannabis, delta-9-tetrahydrocannabinol, cannabinoid, ergogenic, ergolytic

## Abstract

**Purpose:** There is extensive public and scientific interest in the influence of cannabis and the psychoactive cannabinoid, delta-9-tetrahydrocannabinol (THC), on exercise performance. Unfortunately, recent, up-to-date studies are lacking. The aim of the current study was to address the hypothesis that ingestion of edible marijuana, prior to exercise, would have unfavorable effects on the physiological response to exercise and on exercise performance.

**Methods:** 17 Healthy adult male and female habitual exercisers, who were regular users of cannabis products, were screened for study participation. 10 were enrolled, and data from 9 [8 males, 1 female, aged 25±3 years, with peak oxygen uptake of 56.5±11.7 ml/kg/min (mean ± SD)] were retained. Participation included two exercise sessions, each preceded by self-administration and ingestion of either edible marijuana (containing 10 mg THC) or placebo. Cardio-respiratory responses (*via* indirect calorimetry) to stationary cycle ergometer exercise (8 min at 50, 100 and 150 W) were recorded before completion of a 20-min Functional Threshold Power test (FTP^20^) and a sprint test involving maximal effort until volitional fatigue.

**Results:** Edible marijuana increased the concentration of circulating THC and THC metabolites, and evoked sensations of intoxication and altered psychoactive state. Cardio-respiratory responses to staged cycle ergometer exercise were normal and were unaffected by edible marijuana. Compared with placebo, edible marijuana did not influence FTP^20^ (Placebo 253±75 vs THC: 251±72 W (mean±SD); *p* > 0.45) or peak power output during the sprint test (Placebo: 710±201 vs. THC: 732±136 W; *p* = 0.864).

**Conclusion:** 10 mg of THC, when ingested prior to exercise by regular exercisers and habitual users of cannabis, had little effect on the physiological response to standardized cycle ergometer exercise, and was neither ergogenic nor ergolytic.

## Introduction

The use of products derived from cannabis is increasing among the general population, including athletes and habitual exercisers (Docter et al., 2020; Weinberger et al., 2022). While recent consideration of the potential ergogenic benefits of cannabidiol has been reported (Cochrane-Snyman et al., 2021; Isenmann et al., 2021; Crossland et al., 2022; Sahinovic et al., 2022), the influence of products containing the psychoactive cannabinoid, delta-9-tetrahydrocannabinol (THC) is relatively understudied. Some athletes are incorporating THC-containing cannabis products in their routine training (YorkWilliams et al., 2019; Kennedy, 2022; Ogle et al., 2022) despite the lack of up-to-date empirical information as to how cannabis might influence training and exercise performance. In this regard, there are several reviews that have critically evaluated studies on this topic (Kramer et al., 2020; Burr et al., 2021; Charron et al., 2021). A number of features stand out from these reviews, such as, there currently appear to be more review papers describing the acute influence of cannabis on exercise in healthy, disease-free adults than there are original studies. Noteworthy, most of these original studies were completed in the 1970s and 1980s (Steadward and Singh, 1975; Avakian et al., 1979; Bird et al., 1980; Renaud and Cormier, 1986), thus they may not be reflective of contemporary cannabis products, products which are known to contain greater amounts of THC, and are considered to be more potent than older products (Cinnamon Bidwell et al., 2018; ElSohly et al., 2021). In addition to changes in cannabis products, during the previous 40–50 years there have been significant technological advancements that have improved the ease and precision with which exercise physiology parameters may be quantified. Importantly, the recent reviews highlight the need for new investigations to revisit and provide up-to-date insight into the question of the influence of cannabis products on exercise. Specifically, the influence of contemporary preparations of cannabis products on exercise is unknown. Further, no studies have described the influence of modern preparations of edible marijuana on exercise. Moreover, in light of the prevalence of cannabis use among habitual exercisers and athletes, there is a need to understand the influence of edible marijuana on the physiological responses to standardized exercise, and on the performance of endurance and high-intensity, all-out exercise. The current investigation attempts to addresses this need.

Federal, state, and institutional regulations, have made studies of cannabis use in humans notoriously difficult to accomplish, thereby contributing to the paucity of contemporary cannabis-exercise information. We have recently circumvented some of these difficulties and have been able to complete pharmacokinetic and pharmacodynamic studies of cannabis products in humans (Ewell et al., 2021) using a naturalistic/observational approach inspired by a previous study (Bidwell et al., 2020). In this approach, research participants purchased commercially available edible marijuana and self-administered (i.e. ingested) the product off-campus. Thus, investigators did not directly administer cannabis, and cannabis was consumed in an environment consistent with the rules of our academic institution.

The aim of the current study was to determine the acute influence of a cannabis-derived product, commercially available edible marijuana, on the physiological response to standardized exercise, endurance exercise performance, and performance during short-term high-intensity exercise. The rationale for choosing to study an edible product over inhaled cannabis was twofold: first, inhalation of combustible materials exposes the lungs and blood to a variety of potentially toxic substances, such as hydrocarbons, thus edible marijuana avoids the potential confounding influence of inhaled cannabis on oxygen carrying capacity. Second, we have previously described the pharmacokinetics of the specific edible marijuana product to be studied (Ewell et al., 2021), therefore we were in an informed position when making decisions with respect to protocol development and the timing of exercise testing relative to ingestion. Based on published reviews (Kennedy, 2017; Burr et al., 2021; Charron et al., 2021), the hypothesis was that ingestion of edible marijuana prior to exercise would have undesirable effects on the physiological response to exercise and on exercise performance. These undesirable effects would include excessive heart rate, increased rate pressure product (an indicator of myocardial oxygen demand), a decrease in the mean power that could be sustained on a cycle ergometer for 20-min, and decreased tolerance of all-out, high-intensity exercise.

## Materials and methods

### Participants

Healthy, recreationally active males and females aged 21–40 years were invited to participate. Inclusion criteria included regular cannabis use (self-reported use during the previous year ≥12), engagement in a minimum of 30 min of structured exercise at least 5 days a week throughout the previous year, and prior use of a cannabis product containing at least 10 mg of THC without an adverse reaction. Exclusion criteria included previous diagnosis of a bipolar disorder or schizophrenia, heart disease, peripheral vascular disease, high blood pressure, stroke, or a heart murmur, pregnancy or breast feeding, or a physician-identified contra-indication to exercise based on a graded exercise test with 12-lead electrocardiogram (ECG) assessment. This study was approved by the Colorado State University Institutional Review Board (Protocol #2827, approved 30 November 2021). All participants provided written informed consent prior to study commencement. The study was registered with ClinicalTrials.gov (Identifier: NCT05192239).

### Overview of experimental design

This study utilized a randomized, single-blind, placebo-controlled crossover design. Following screening and two habituation visits, participants reported to our laboratory on two separate occasions to complete stationary cycle ergometer exercise after ingesting either placebo or edible marijuana. Stationary cycle ergometer exercise comprised three protocols performed within a single session: (1) three consecutive standardized bouts, each lasting 8-min, at work rates of 50, 100, and 150 W; (2) a test of endurance: the 20-min functional threshold power test (FTP^20^); and (3) a sprint test involving maximal effort until volitional fatigue. Indirect calorimetry and heart rate measurements were used to determine the cardio-respiratory responses to the standardized exercise bouts, and arterialized-venous blood was collected for analysis of circulating concentrations of lactate, THC, and THC metabolites.

### Screening

Potential research participants reported to the laboratory for an initial screening visit that consisted of a medical history/screening questionnaire, and assessment of body composition and peak oxygen uptake (VO_2peak_). Body composition assessment comprised measurements of height (stadiometer), body mass (physician’s scale), and use of dual-energy x-ray absorptiometry (Hologic, Discovery W, QDR Series, Bedford, Massachusetts, United States), as previously described (Williams et al., 2021). VO_2peak_ was assessed during incremental cycle ergometer exercise (25–35 W/min) to voluntary fatigue using an electrically braked ergometer (Corvial Cpet, Lode BV, Groningen, Netherlands), and indirect calorimetry (ParvoMedics TrueOne 2400; Salt Lake City, Utah), as previously described (Newman et al., 2019). Participants were instructed to maintain a pedal cadence between 60 and 90 rpm. Fatigue was defined as an inability to maintain a pedal cadence of 60 rpm. VO_2peak_ was recorded as the greatest value for VO_2_ averaged over 30-s. Prior to, during and following exercise, beat-to-beat heart rate was recorded using 12-lead ECG and the cardiograms were inspected by a physician for presence of contraindications to exercise.

### Habituation

To familiarize participants with the exercise protocols, two habituation sessions were completed. These sessions were almost identical to the data collection visits described below; blood collection, indirect calorimetry, and placebo/marijuana ingestion were not included in the habituation sessions.

### Placebo and edible marijuana

At the time of data collection, investigators not in possession of appropriate licensing by the Drug Enforcement Agency of the United States of America were not permitted to procure and administer products containing THC for the purposes of scientific research in humans. Accordingly, research participants were instructed to purchase their own edible marijuana products from a local distributor identified by the research team. In a previous study (Ewell et al., 2021), we have characterized the pharmacokinetics of commercially available edible marijuana (Ripple Blood Orange Gummies, Stillwater Brands, Commerce City, Colorado, United States); participants were instructed to purchase this edible marijuana product. All participants provided proof of purchase (receipts) and unopened packaging for inspection by the research team. The dose of THC to be self-administered was 10 mg, ingested as two gummies.

The placebo was a commercially available, THC-free product (Welch’s Fruit Snacks, Park Ridge, New Jersey, United States). The energy content of the placebo and edible marijuana gummies was similar (within 5 kcal).

### Data collection visits

The time of day for the data collection visits was kept constant for each participant (within 30-min). To standardize pre-exercise nutrition, participants were provided with a commercially available liquid meal (Ensure Original Meal Chocolate Nutrition Shake; Abbott Laboratories, Chicago, Illinois) and a snack (Kind Bar, Dark Chocolate Nuts and Sea Salt; New York, New York). The total energy value of the pre-exercise nutrition was 1,674 kJ (400 kcal) and comprised 21g fat, 48 g carbohydrate, and 15 g protein. The pre-exercise nutrition was consumed 60-min prior to the commencement of the study procedures.

At the time of data collection, products containing THC were not permitted within the grounds of our university campus, thus the initial procedures were completed at an off-campus venue. Participants were collected, *via* motor vehicle, from their personal residence and were transported to the off-campus venue for measurement of heart rate and blood pressure, and collection of ∼10 ml of venous blood from an antecubital vein. The blood was to be subsequently analyzed for concentrations of THC and THC metabolites to provide additional support for the participant’s verbal confirmation that they had abstained from THC during the previous 96-h.

To facilitate the blinding of participants to treatments (i.e. placebo and edible marijuana), two envelopes were provided. The envelopes were labeled “A” and “B” and were presented face-down, thereby ensuring the participants were unable to see the labels. Participants placed and sealed a single dose of edible marijuana (10 mg of THC; 2 gummies) in one envelope, an investigator placed and sealed the placebo in the other envelope. Throughout the procedure, the participants remained naïve as to the labeling (A vs. B) and specific contents (placebo vs. edible marijuana) of the individual envelopes.

Following collection of baseline data and venous blood, participants ingested the gummies from either Envelope A or Envelope B. The treatment order was randomized. The unopened envelope remained sealed until the subsequent visit and was stored with the personal possessions of the research participants; this ensured that members of the research team were never in contact with the edible marijuana.

Immediately after placebo/marijuana ingestion, participants were transported, by motor vehicle, to the laboratory. The duration of the journey was approximately 5-min. On arrival at the laboratory, a venous catheter was placed in a dorsal hand vein, and the hand and wrist were wrapped in an electric heated blanket for subsequent sampling of arterialized-venous blood (Forster et al., 1972).

Based on previous research (Ewell et al., 2021), the mean time to peak circulating THC concentration after ingesting the edible marijuana was approximately 35-min. Accordingly, 35-min after placebo/marijuana ingestion, stationary cycle ergometer exercise began. Three separate protocols were completed during each laboratory visit; each protocol was separated by 5-min of recovery. The first was designed to provide an opportunity for studying the physiological responses to standardized exercise. The second involved a test of endurance performance. The third protocol examined high-intensity exercise performance to task failure. An air-braked stationary cycle ergometer was used (Concept2 BikeErg, Concept2 Inc., Morristown, Vermont, United States) for all three protocols. Calibration was undertaken as per the manufacturer’s guidelines. Power outputs were recorded electronically using software provided by the ergometer manufacturer (ErgData, Concept2 Inc., Morristown, Vermont, United States).

During Protocol 1, participants were instructed to cycle at power outputs of 50, 100 and 150 W for 8 min per stage. Air resistance (damper setting) was self-selected by each participant during the habituation sessions and was kept constant (within participants, and between trials) throughout the standardized exercise protocol. To facilitate steady-state data collection, expired gases were analyzed during the final 4 min of each 8-min stage *via* indirect calorimetry (ParvoMedics TrueOne 2400; Salt Lake City, Utah). Additionally, during the final 30-s of each stage, approximately 2-ml of arterialized-venous blood was sampled for measurement of circulating lactate concentration, rating of perceived exertion was determined *via* the Borg Scale (range 6-to-20) (Borg, 1982), heart rate was determined *via* short-range telemetry (Polar T31, Bethpage, New York, United States), and blood pressure was assessed *via* manual auscultation. Rate pressure product, an indicator of myocardial oxygen consumption, was calculated as the product of heart rate and systolic blood pressure.

For Protocol 2, participants completed an FTP^20^ (Mackey and Horner, 2021). This test involved assessment of the maximal mean power that could be sustained for 20-min. The FTP^20^ is considered a predictor of cycling endurance performance, and is commonly used in laboratories and by cyclists of varied abilities to gauge performance and training status (Mackey and Horner, 2021). Immediately prior to and following the FTP^20^, approximately 2-ml of arterialized-venous blood was collected for measurement of circulating lactate concentration, and ∼10 ml was collected for measurement of concentrations of THC and THC metabolites. RPE and heart rate were recorded at minutes 4, 8, 12, 16 and 20. To facilitate a maximal effort, and to remove any potential ventilatory burden, expired gases were not collected during the FTP^20^.

During the final protocol, participants were given 10-s to gradually increase their pedal revolutions to a maximal cadence. During this period the air-resistance was at the lowest setting (i.e. damper setting 1). At 10-s the air-resistance was set to maximum (i.e. damper setting 10) and participants maintained their maximal cadence until task failure, defined as a pedal cadence falling below 70 rpm. This “all-out” effort was considered as an indicator of capacity for high-intensity exercise. Immediately prior to and 5-min following the completion of the test, approximately 2-ml of arterialized-venous blood was sampled for measurement of circulating lactate concentration. At the end of the study session, approximately 75 min after ingestion of placebo/marijuana, blood was also sampled for measurement of THC and THC metabolites.

To obtain insight as to participants’ perceptions of altered psychotropic state, throughout each of these data collection visits participants completed several simple self-report measurements that addressed their perceived degree of intoxication and symptoms of cannabis use. These questionnaires were based on tools used in previous studies (Bidwell et al., 2018; Bidwell et al., 2020). The questionnaires required responses submitted *via* visual analog scales (VAS), and were administered at baseline, 30 min after baseline (pre-standardized exercise), and following every exercise protocol thereafter. Specifically, the participants were to respond to questions pertaining to their perceptions of physical, mental, and overall degree of intoxication.

### Blood processing and analysis

Blood collected for analysis of lactate concentration was immediately transferred to chilled tubes coated with sodium fluoride/potassium oxalate and placed on ice. Within an hour of blood collection, lactate was determined using an automated analyzer (YSI 2900, Xylem Inc; White Plains, New York). Blood intended for analysis of concentrations of THC, and THC metabolites was immediately transferred to chilled tubes coated in K3 Ethylenediaminetetraacetic acid and placed on ice. Within an hour of blood collection, plasma was isolated from whole blood after chilled (4°C) centrifugation. Aliquots of.

Plasma (1 ml) were then placed in frozen storage (−80°C) until subsequent analysis. Concentrations of THC and the THC metabolites, 11-hydroxytetrahydrocannabinol (THC-OH) and 11-nor-9-carboxytetrahydrocannabinol (THC-COOH) were determined using established protocols involving liquid chromatography and tandem mass spectrometry (LC-MS/MS), as previously described in detail (Ewell et al., 2021). Noteworthy, the research team members responsible for these analyses remained naïve as to the timing and conditions (i.e. placebo vs. marijuana) under which the samples were collected.

### Statistical analysis

All data, unless otherwise stated, are expressed as mean and standard deviation. To compare baseline values between placebo and edible marijuana for circulating concentrations of THC, and THC metabolites, one-way analysis of variance (placebo vs edible marijuana) was used, unless a non-parametric equivalent (i.e. one way analysis of variance on ranks) was required on account of unequal variance. The standardized exercise statistical analysis was conducted in R (R Core Team; Vienna, Austria) as linear mixed models using the Lmer and LmerTest packages (Bates et al., 2015), and Emmeans package was used for post hoc Tukey testing to explore main effects and interactions. Time and Condition were considered fixed effects, whereas Subject was considered a random effect to account for the repeated measures. Unlike an electrically braked ergometer, it is not feasible to externally fix the work rate on an air braked ergometer; the actual work rate is determined by the damper setting and the participant’s pedal cadence. During the standardized exercise test, participants were instructed to exercise at 50, 100 and 150 W. Accordingly, for statistical analysis of these data, work rate error, defined as the difference between the prescribed work rate and the actual work rate, was considered as a covariate in our model. One- and two-way analysis of variance, with repeated measures, were employed to detect differences among variables during the performance of the FTP^20^ and in the sprint to fatigue tests using SigmaPlot 14.5 (Systat Software Inc., San Jose, California). Specifically, one-way repeated measures ANOVA was used to compare mean work rate (placebo vs edible marijuana). Two-way ANOVA with repeated measures (time) was used to compare power outputs across consecutive stages (placebo vs edible marijuana). One-way repeated measures ANOVA was used to compare mean power, peak power, time to fatigue, and total work done (placebo vs edible marijuana) during the sprint to fatigue tests. Significance was defined as *p* < 0.05. Wherever a significant difference was detected, a post-hoc Tukey test was utilized to further explore these differences.

## Results

### Participants

**FIGURE 1:**
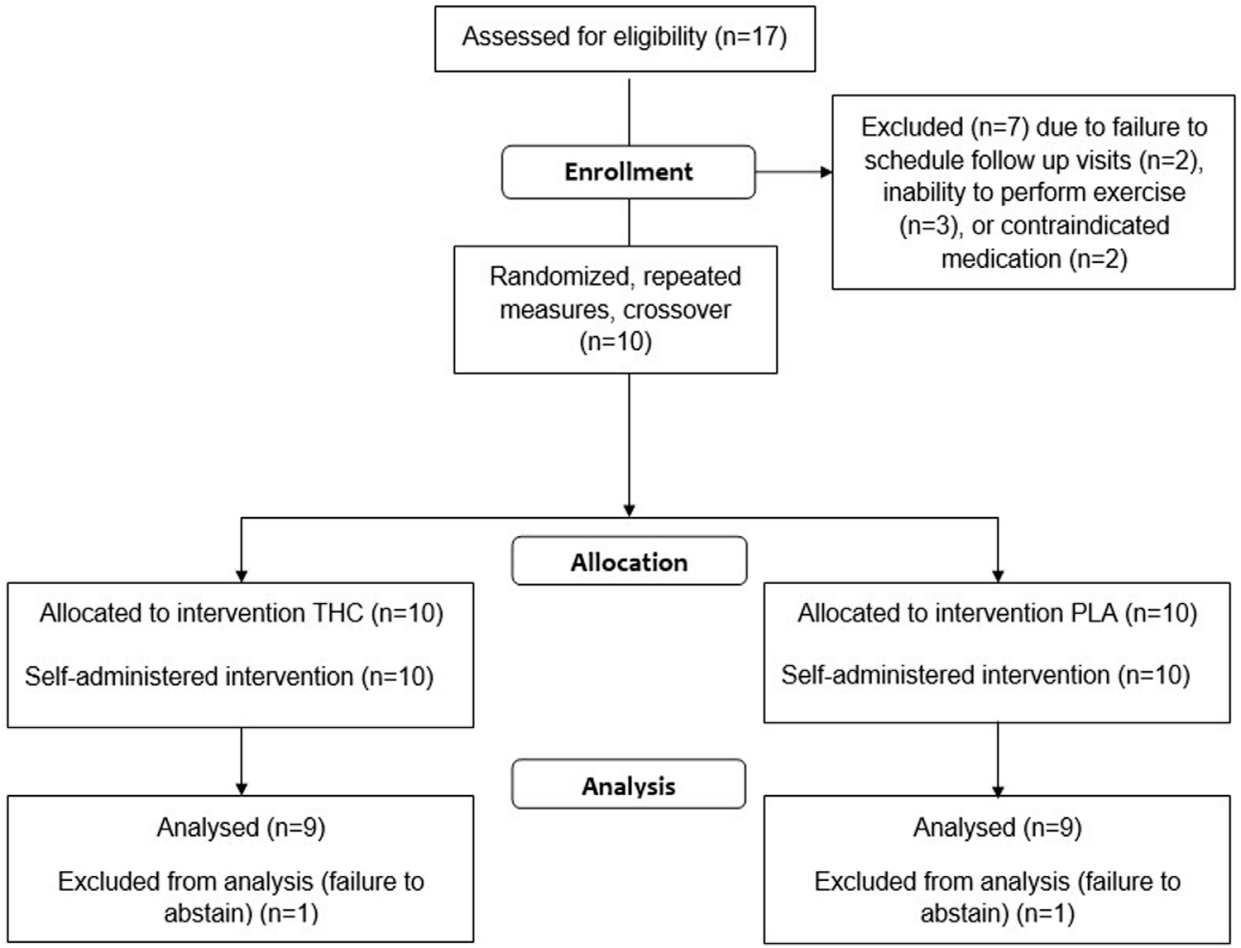
Consolidated Standards of Reporting Trials (CONSORT) flow diagram.

The flow of participants from initial screening to study completion is depicted in the Consolidated Standards of Reporting Trials (CONSORT) flow diagram (Figure 1). Seventeen adults were assessed for eligibility. Two adults declined invitations to schedule post-screening study visits, two were excluded from participation on account of use of contraindicated medications, and three were discontinued during the habituation visits as they were unable to sustain the required work rate. Thus, ten participants completed all study protocols, however, data from one participant were excluded from the final analysis. The rationale for this exclusion was high baseline circulating concentrations of THC (6.95 ng/ml) and THC metabolites (THC-COOH: 222 ng/ml, THC-OH: 2.93 ng/ml), indicating failure to abstain from THC use within the 96-h prior to testing. Accordingly, data from nine participants are presented. Selected physiological characteristics are presented in Table 1 and appear typical for young, habitual exercisers. Participants reported habitual frequency of use of cannabis products as 12 ± 12 uses per month (Range: 1–30 uses per month).

**TABLE 1:** Selected physiological characteristics of study participants.

Variable	Mean ± SD	Range
Sex (M/F)	8/1	−
Age (years)	25 ± 3	21–29
Height (cm)	167.3 ± 25.5	103.0–190.5
Body Mass (kg)	75.97 ± 13.30	53.05–100.51
Lean Mass (kg)	57.98 ± 9.79	37.9–72.8
Fat Mass (kg)	15.32 ± 3.99	11.46–24.49
Body Fat (%)	20.12 ± 3.05	16.70–25.10
VO_2peak_ (L/min)	4.34 ± 1.26	2.09–6.24
VO_2peak_ (ml/kg/min)	56.5 ± 11.7	39.8–74.8

VO_2peak_: Peak oxygen uptake.

### THC, THC metabolites, and visual analogue scale scores

**FIGURE 2:**
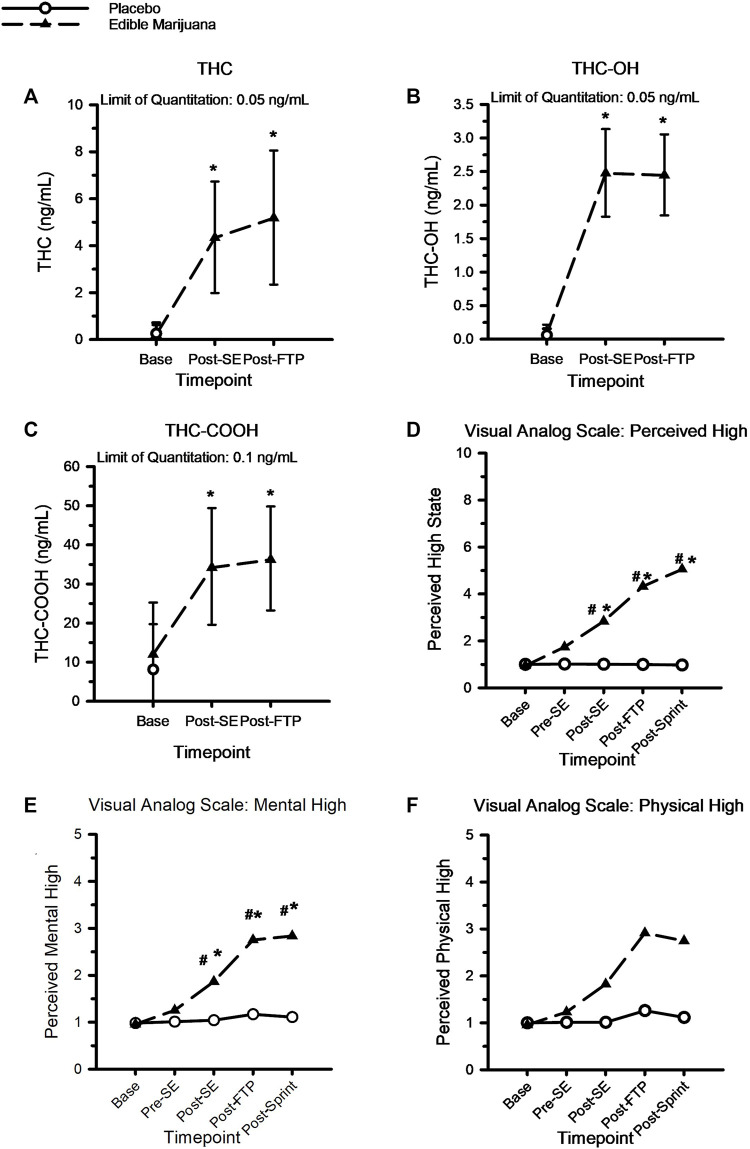
A-to-C: Circulating concentration of THC **(A)**, THC-OH **(B)** and THC-COOH **(C)** following ingestion of edible marijuana (10 mg of THC). Data are mean and SD. D-to-F, Visual analog scores of perceived intoxication specific to “High” **(D)**, Mental High **(E)** and Physical High **(F)** following ingestion of edible marijuana and placebo. For all figures, * depicts different from baseline (*p* < 0.05), # depicts different between conditions (*p* < 0.05). For the visual analog figures, error bars have been omitted for clarity.

Figure 2 shows the circulating concentrations of THC, THC metabolites, and VAS scores pertaining to perceptions of intoxication from the edible marijuana. There were no differences at baseline for circulating THC (Placebo: 0.3 ± 0.4 vs. Edible marijuana: 0.2 ± 0.4 ng/ml; *p* = 1.00), THC-OH (Placebo: 0.1 ± 0.1 vs. Edible marijuana: 0.1 ± 0.1 ng/ml; *p* = 1.00), THC-COOH (Placebo: 8.5 ± 11.3 vs. Edible marijuana: 12.3 ± 12.9 ng/ml; *p* = 0.39), or for any of the VAS scores between conditions (Placebo vs. Edible marijuana; all *p* > 0.3). Following ingestion of edible marijuana, circulating concentrations of THC and THC metabolites were increased above baseline at all subsequent time points (*p* < 0.001). Concentrations were not different between completion of standardized exercise and the FTP^20^ (all *p* > 0.58).

Compared with baseline and placebo, ingestion of edible marijuana increased perceptions of feeling “mentally stoned” and “high” following the standardized exercise bout; these perceptions remained greater than baseline throughout the duration of the study visit (*p* < 0.05). However, perceptions of feeling “physically stoned” were not different from baseline (*p* = 0.42) or between placebo and edible marijuana (*p* = 0.34).

### Physiological responses to standardized exercise

**FIGURE 3:**
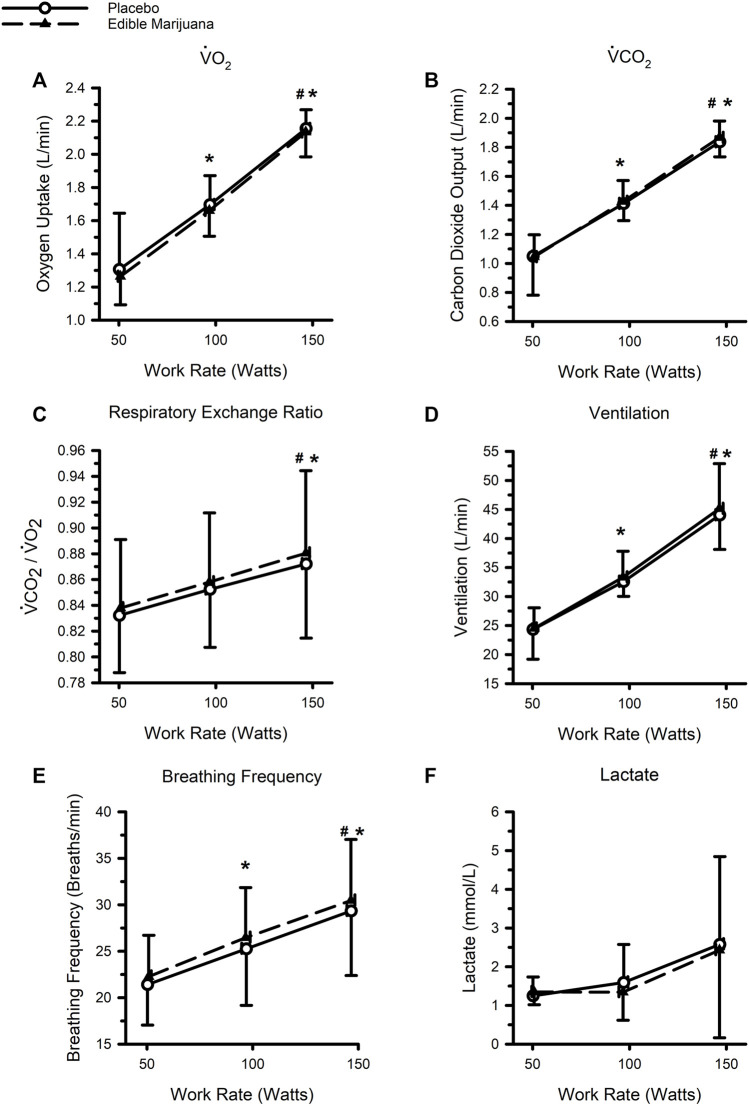
Ventilatory and metabolic responses to standardized stationary cycle ergometer exercise following ingestion of edible marijuana (10 mg THC) or placebo: Oxygen uptake (V̇O_2_) **(A)**, Carbon dioxide output (V̇CO_2_) **(B)**, Respiratory exchange ratio **(C)**, Ventilation **(D)**, Breathing Frequency **(E)**, and Circulating concentrations of lactate **(F)**. For all figures, data are presented as mean and SD. * depicts different from the 50 W (main effect of work rate; *p* < 0.05), # depicts different from 100 W (main effect of work rate; *p* < 0.05).

The physiological responses to standardized exercise are presented in Figure 3. As work rate increased, so did VO_2_, carbon dioxide production, ventilation, and breathing frequency (all *p* < 0.05); respiratory exchange ratio was greater at 150 W compared with 50 and 100 W, but not different between 50 and 100 W. Ingestion of edible marijuana did not influence VO_2_, carbon dioxide production, ventilation, or respiratory exchange ratio (all *p* > 0.6). Similarly, ingestion of edible marijuana did not influence breathing frequency (*p* = 0.08). Blood lactate concentration was not appreciably different across the three work rates, nor was it influenced by edible marijuana. Heart rate, systolic and diastolic blood pressures, rate pressure product, and rating of perceived exertion are presented in Table 2. All variables increased with increasing work rate (main effect: all *p* < 0.002) but were not influenced by edible marijuana (Interaction: all *p* > 0.1).

**TABLE 2:** Cardiovascular responses and ratings of perceived exertion during standardized stationary cycle ergometer exercise following ingestion of edible marijuana or placebo.

Variable	Plac THC	Work rate (watts)				ANOVA *p*-values main effects		
		0	50	100	150	Work rate	Plac THC	X
**Heart Rate (beat/min)**	Plac	66±16	95±14	108±18	130±26	<0.001	0.109	0.105
	THC	63±13	97±15	113±20	135±27			
**SBP (mmHg)**	Plac	127±13	138±8	151±9	162±11	<0.001	0.134	0.151
	THC	129±10	135±10	146±8	154±13			
**DBP (mmHg)**	Plac	71±5	77±8	80±7	77±7	0.002	0.381	0.467
	THC	72±8	82±9	81±9	77±8			
**RPP (mmHg• (beat/min))**	Plac	8135 ± 1770	13,042 ± 1766	16,371±3099	21,074 ± 4793	<0.001	0.817	0.778
	THC	8141 ± 1838	13,050 ± 2088	16,535 ± 3039	20,859 ± 4877			
**RPE**	Plac	6±0	8±1	11±1	12±1	<0.001	0.358	0.409
	THC	6±0	8±1	10±1	12±1			

Data: mean and SD, Plac: Placebo. THC: Edible marijuana (10 mg of delta-9-tetrahydrocannabinol). SBP: Systolic blood pressure. DBP: Diastolic blood pressure. RPP: Rate pressure product. RPE: Rating of perceived exertion. ANOVA: Analysis of variance. X: statistical interaction.

### Functional threshold power test

**FIGURE 4:**
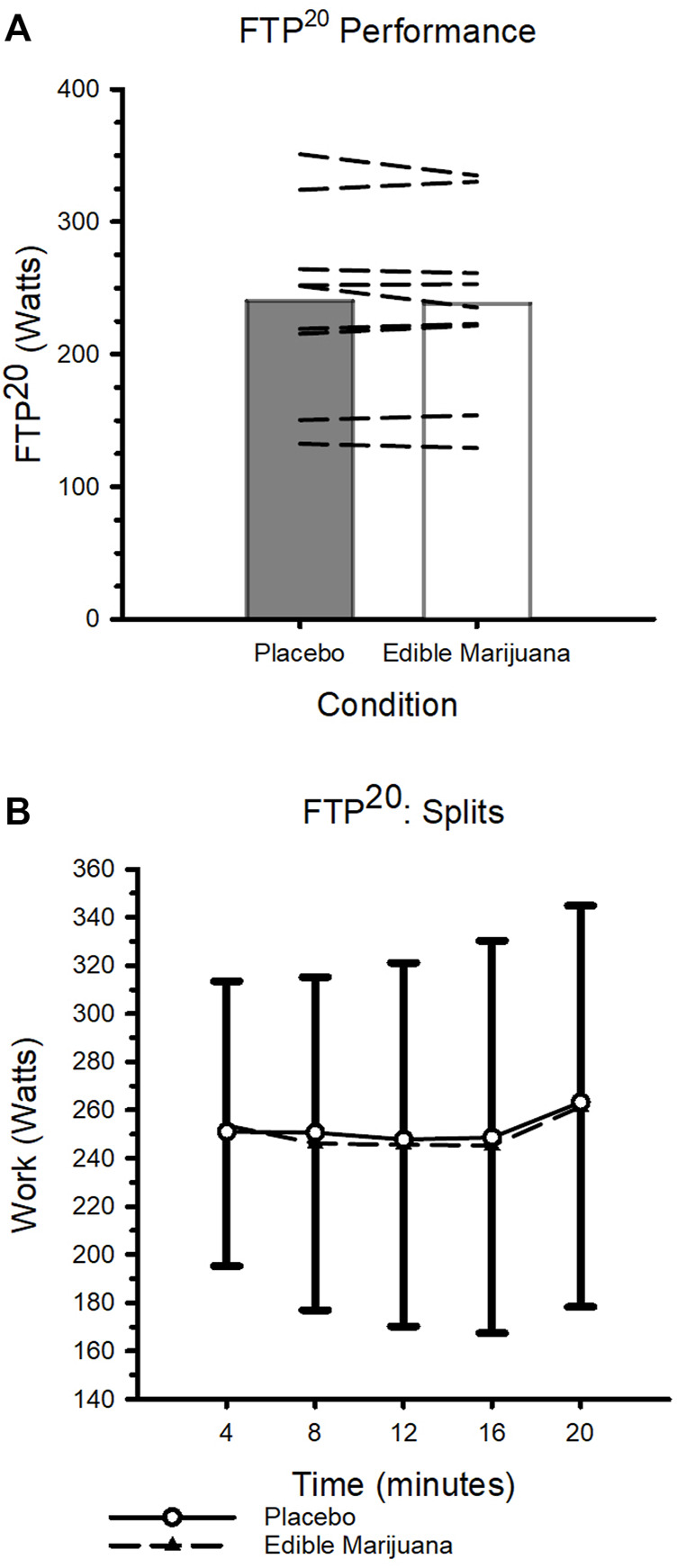
FTP^20^ Performance after ingestion of edible marijuana (10 mg THC) or placebo **(A)** Maximal sustained mean power output. Bars represent mean data; dashed lines represent individual responses. *p* = 0.454. **(B)** FTP^20^ performance depicted over 4-min intervals. Data are presented as mean ± SD.

Data collected from the FTP^20^ are presented in Figure 4. Neither mean work rate nor power outputs during consecutive 4-min segments (indicative of pacing strategy) were influenced by ingestion of edible marijuana (both *p* ≥ 0.454). Throughout the test, heart rate and ratings of perceived exertion were increased (both *p* < 0.01; Table 3), however ingestion of edible marijuana did not influence either of these variables. Similarly, blood lactate concentration was greater (*p* = 0.001) following completion of the test compared with baseline, but end-exercise lactate concentration was not influenced by ingestion of edible marijuana (Placebo vs. edible marijuana: 6.9 ± 1.9 vs. 6.5 ± 2.4 mmol/L; *p* = 0.34).

**TABLE 3:** Heart rate and ratings of perceived exertion during performance of the 20-min functional threshold power test (FTP^20^) after ingestion of edible marijuana or placebo.

Variable	Time (min)	Plac	THC	ANOVA *p*-values: Main effects		
				Time	Plac THC	X
Heart Rate (beat/min)	0	91 ± 21	100 ± 17	< 0.01	0.087	0.465
	4	156 ± 14	162 ± 15			
	8	165 ± 12	169 ± 13			
	12	169 ± 11	173 ± 12			
	16	171 ± 12	173 ± 11			
	20	178 ± 10	180 ± 12			
RPE	0	−	−	< 0.01	0.320	0.604
	4	15 ± 2	15 ± 1			
	8	17 ± 2	17 ± 1			
	12	18 ± 1	18 ± 2			
	16	18 ± 1	18 ± 1			
	20	19 ± 1	20 ± 1			

Data: mean and SD, Plac: Placebo. THC: Edible marijuana (10 mg of delta-9-tetrahydrocannabinol). RPE: Rating of perceived exertion. ANOVA: Analysis of variance. X: statistical interaction.

### High-intensity exercise

**FIGURE 5:**
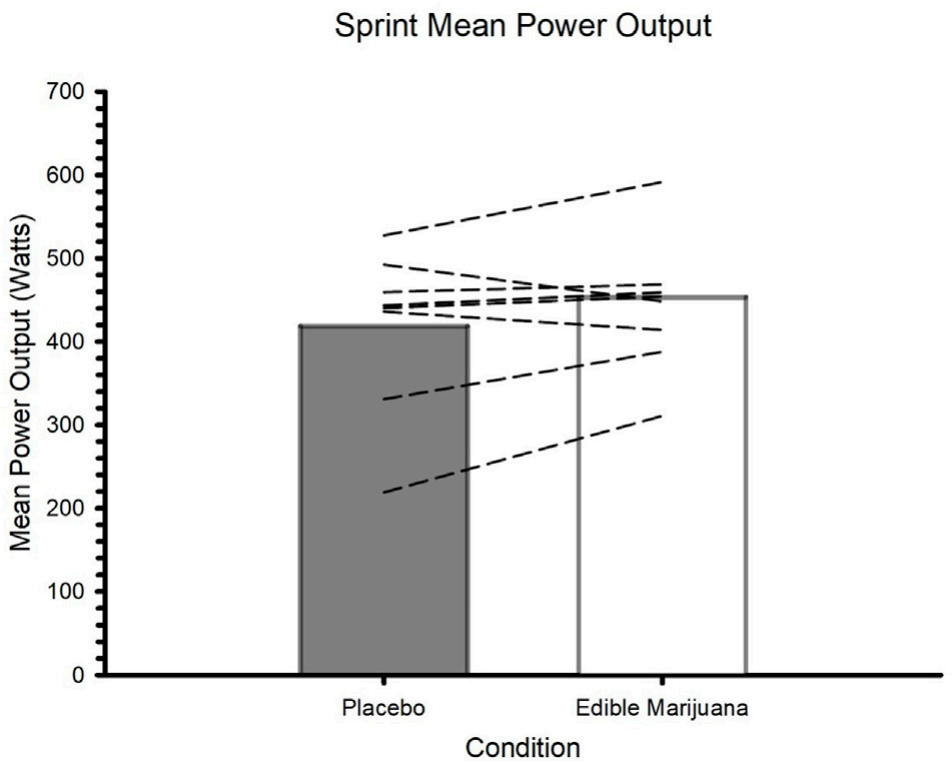
Mean power during maximal effort test to volitional fatigue after ingestion of edible marijuana (10 mg THC) or placebo. Bars represent mean data; dashed lines represent individual responses. *p* = 0.173.

Edible marijuana did not influence any of the outcome variables collected during the trial involving maximal effort to volitional fatigue. These variables included mean power (Figure 5; placebo vs. edible marijuana; 422 ± 92 vs. 454 ± 77 W, *p =* 0.173), peak power (710 ± 201 vs. 732 ± 136 W, *p* = 0.864), time to fatigue (43 ± 33 vs. 43 ± 32 s, *p* = 0.835), and total work done (20.14 ± 14.91 vs. 19.65 ± 14.50 kJ, *p* = 0.865). Blood lactate concentration was greater 5 minutes following the sprint compared to immediately prior (*p =* 0.034); ingestion of edible marijuana did not influence end-exercise lactate (6.7 ± 1.8 vs. 7.3 ± 2.0 mmol/L; *p =* 0.771).

## Discussion

To our knowledge, the current study represents the first investigation in approximately 40–50 years to describe the influence of cannabis derived products on the physiological response to exercise and on exercise performance in healthy, disease-free, habitual exercisers. Moreover, this is the first study to investigate the potential influence of modern formulations edible marijuana on exercise. The specific formulation under investigation was a commercially available product reflective of contemporary preparations of THC. Our data suggest that edible marijuana, when ingested prior to exercise by regular users of cannabis products, had little effect on the physiological response to standardized cycle ergometer exercise, and was neither ergogenic nor ergolytic.

The influence of cannabis products on exercise and their use in sports has been extensively reviewed (Campos et al., 2003; Huestis et al., 2011; Kennedy, 2017; Ware et al., 2018; Docter et al., 2020; Kramer et al., 2020; Burr et al., 2021; Charron et al., 2021); readers are referred to these publications for detailed description and thorough critical analysis. In general, our data are consistent with previous studies and reviews that have concluded that the acute influence of cannabis products on responses to exercise and exercise performance is *mostly* modest and/or unremarkable. Exceptions should be noted. For example, some have reported increased heart rate, systolic blood pressure, and breathing frequency following cannabis use (Steadward and Singh, 1975; Avakian et al., 1979; Renaud and Cormier, 1986). Several possible and not necessarily mutually exclusive explanations could account for the apparent differences between these previous studies and the observations reported in the current study. These include dose, timing and method of THC administration, and the health of the research participants. Each of these explanations shall be discussed in the paragraphs that follow.

As recently reviewed (Charron et al., 2021), previous acute studies of cannabis and human performance have used vastly different drug protocols incorporating a large range of both THC doses (∼7–70 mg) and durations of time between cannabis consumption and initiation of exercise testing (20–100 min). Further complicating data interpretation, a variety of methods of THC administration have been used, including inhalation of combusted or vaporized products, and orally ingested products. The pharmacokinetics and resultant pharmacodynamics of cannabinoids are notoriously variable (Millar et al., 2018; Poyatos et al., 2020; Ewell et al., 2021; Williams et al., 2021), thus it is highly plausible that inconsistent observations could be, at least in part, attributed to different circulating and tissue concentrations of THC at the time of testing. In the current study, a previously described edible commercial product, reflective of contemporary THC preparation, was used (Ewell et al., 2021). The THC dose of 10 mg was sufficient to induce sensations of intoxication and altered psychotropic state, without compromising necessary motor functions and leading to a loss of balance and increased dizziness, as has been previously reported (Bird et al., 1980; Whiting et al., 2015). Further, the THC dose evoked quantifiable increases in circulating concentrations of THC and THC metabolites that compared well with previous reports (Ewell et al., 2021). Importantly, the timing of exercise initiation was dictated by the previously determined time to maximal circulating THC concentration (Ewell et al., 2021), and our data indicate that circulating THC concentration remained elevated until the end of the study session (Figure 2). This continued elevation is unsurprising in light of the product’s reported half-life: ∼268 min (Ewell et al., 2021).

With respect to our rationale for choosing to study an edible product over inhaled cannabis, inhalation of combustible materials exposes the lungs and blood to a variety of potentially toxic substances, such as hydrocarbons. In addition, inhaling combustible cannabis can lead to variability in THC bioavailability on account of differences in tidal volume, duration of held inspiration, and heterogenous distribution of THC within cannabis cigarettes. Thus, use of edible marijuana avoided the potential confounding influence of inhaled cannabis on oxygen carrying capacity and facilitated consumption of a standardized (less variable) THC dose.

Several of the previous human studies of THC consumption and exercise have recruited research participants with clinical conditions and chronic diseases, including angina (Aronow and Cassidy, 1974, 1975), and chronic obstructive pulmonary disease (Abdallah et al., 2018), and/or people who were not regular exercisers (Avakian et al., 1979). In the current study, although the degree of conditioning spanned a broad range (VO_2peak_: ∼40–75 ml/kg/min; Table 1) all participants were free from chronic disease and reported participating in a minimum of 150 min of exercise per week during the previous 12-month. Thus, our data are most relevant to young, recreationally active adults and amateur athletes. It is plausible that the acute influence of THC on exercise may be modified by the presence of a chronic disease, or by elite/professional athletic status. For example, the ergolytic effect of THC previously reported in patients with angina (Aronow and Cassidy, 1974, 1975) has been attributed to increased myocardial oxygen consumption (Burr et al., 2021). In the current study, the potential additional cardiovascular demand of THC may have been trivial for a group of healthy, habitual exercisers, hence the absence of an ergolytic effect. In addition, many of the cycle ergometer work rates generated in previous THC studies involving non-exercisers and people with chronic diseases are relatively low (<200 W), but elite cyclists are capable of performing at work rates in excess of 400 W (Clark and Macdermid, 2021). Thus, conclusions regarding the influence of THC in non-exercisers and people with chronic diseases are likely to have limited translational relevance for elite/professional athletes. In one review, this translational relevance was characterized as absurd (Burr et al., 2021).

There are several limitations of the current study. First, self-reported frequency of cannabis use by our research participants was highly variable (range: 1–30 uses per month). A potential additional source of variability within our exercise data could be attributed to differences in regular cannabis exposure, as pharmacokinetics, tolerance, and heart rate have been reported to be influenced by cannabis use history, including age at first cannabis exposure, number of years of cannabis use, and frequency of use (Nowlan and Cohen, 1977; Kirk and de Wit, 1999; Berl et al., 2022). In our study, frequency of use did not predict the magnitude of change in exercise or performance response following edible marijuana ingestion. Unfortunately, data pertaining to age at first use and duration of use were not collected. Another study limitation pertains to our attempts to make the intervention single-blind. Despite the labeling of the envelopes containing the edible marijuana and placebo (e.g. A and B), all participants successfully identified the edible marijuana on account of the resulting altered perceived state of intoxication. In light of the psychoactive properties of edible marijuana, keeping participants naïve as to treatments and placebo appears to be a difficulty unique to this line of research. An additional limitation pertains to the legitimate concern regarding studies that report on the absence of an effect of an intervention and the possibility of insufficient statistical power. In this regard, although our conclusions are based on data collected from nine study participants, we believe they are a true reflection of the unremarkable influence of edible marijuana on exercise performance. To illustrate, based on our FTP^20^ data (where the mean difference between placebo and edible marijuana was 1.94 W, the standard deviation of change was 8.74 W, the pooled standard deviation was 68.0 W, and the r2 was 0.986) with a desired power of 0.80, and an alpha of 0.05, if edible marijuana was to have a statistically significant effect, a sample size of 162 would be required to detect this difference. In contrast, consider the ergogenic effects of established interventions such as caffeine, sodium bicarbonate or dietary nitrate. These are acute interventions that have been shown to evoke physiologically relevant improvements in exercise performance, often with study populations totaling only a handful of participants. Accordingly, we are of the opinion that the requirement of 162 participants to detect a statistical change smaller than 2 W over 20-min implies that the influence of edible marijuana on endurance exercise is essentially non-existent. Finally, recent studies suggest that the prevalence of use of THC-containing products is increasing, presumably on account of relaxation of legal laws and increased accessibility; this increased use is also reflected in populations comprising athletes and habitual exercisers (YorkWilliams et al., 2019; Docter et al., 2020; Kennedy, 2022; Ogle et al., 2022; Weinberger et al., 2022). While our data are specific to the acute physiological influence of THC during exercise, some athletes report using cannabis products for different reasons (Ogle et al., 2022), including increased mental focus, enhanced body awareness, improved observation and awareness of surrounding, promotion of recovery, treatment of injury, attenuation of symptoms of anxiety, and facilitation of sleep. Clearly these uses fall outside the remit of the current study and may benefit from further scientific exploration.

In summary, in light of considerable public interest, and in response to calls and recommendations for up-to-date studies describing the influence of cannabis products on exercise, we are the first to report on the influence of a contemporary preparation of edible marijuana in healthy, disease-free, habitual exercisers. Our data suggest that 10 mg of THC, when ingested by regular users of cannabis products prior to exercise, had little effect on the physiological response to standardized cycle ergometer exercise, and was neither ergogenic nor ergolytic.

## Data Availability

The raw data supporting the conclusion of this article will be made available by the authors, without undue reservation.
